# Supplementary material for: Distributed representations of behaviour-derived object dimensions in the human visual system
Source: Nat Hum Behav. 2024 Sep 9;8(11):2179–93. doi: 10.1038/s41562-024-01980-y (PMC11576512; doi:10.1038/s41562-024-01980-y)
Supplement: Supplementary file 1 — Supplementary Methods 1–3 and Figs. 1–8. [file 41562_2024_1980_MOESM1_ESM.pdf]

nature human behaviour

Supplementary information https://doi.org/10.1038/s41562-024-01980-y

Distributed representations of behaviour-derived object dimensions in the human visual system

In the format provided by the authors and unedited

Supplementary Information

Supplementary Methods

Supplementary Methods 1: Replication in BOLD5000

In order to test how well our results replicate in an independent dataset, we applied our encoding model of behavior-derived object dimensions to a different openly available large-scale fMRI dataset of visual responses. We chose the BOLD5000 dataset [1] because it included object images taken from a different image database, ImageNet [2], as well as scene images taken from the MS CoCo database [3] that consist of a mixture of individual objects and multiple objects in a scene. This allowed us to evaluate the extent to which our results generalize to different participants and images classes. However, we excluded responses to scene categories from the SUN database [4] since these images were specifically designed to exclude objects. We further excluded participant CSI4 from this analysis since they performed only part of the experiment. The resulting dataset comprised three participants who each saw 3,916 images (1,916 images from ImageNet and 2,000 images from MS CoCo).

We constructed our model of behaviorally-relevant object representation for the BOLD5000 stimuli by obtaining predicted dimension values based on the same procedure which we used for the THINGS images (see Methods section *Behavioral embedding*). We then fit an encoding model to each individual participant where we predict voxel-wise responses to each image based on the 66 object dimensions. Mirroring our main analysis, we fit this model in two different ways, 1) in order to evaluate its overall prediction performance, and 2) to evaluate the contribution to individual dimensions in each voxel. To estimate the prediction accuracy of the entire model, we used a simple cross-validated linear regression analogous to our main analysis (Methods section *Linear Regression on fMRI single trial estimates*). We normalized this prediction accuracy based on noise ceilings which we obtained with the same procedure that was used for the THINGS-fMRI dataset [5]. To obtain robust estimates for the contribution of individual dimensions for this prediction, we fit our encoding model again using fractional ridge regression [6]. We determined the best-fitting regularization parameter for each voxel based on the same parameter grid and cross-validation procedure which we used in our parametric modulation model, except it was applied to discrete response estimates instead of time series data (see Methods section *Parametric modulation on fMRI time series*). Finally, to visualize the spatial extent of the model prediction accuracy and the dimension-wise tuning maps, we visualized the results on cortical flat maps [7].

Supplementary Methods 2: Variance partitioning of object shape vs. behavior-derived dimensions

In an exploratory analysis, we tested how much variance in neural responses can be explained by object shape relative to the behavior-derived object dimensions. To this end, we used an image-computable model of object shape and compared its explanatory power to our behavior-derived dimensions using variance partitioning.

We first obtained a model of object shape for all stimuli presented in THINGS-fMRI. To this end, we automatically segmented all images using Segment Anything [8] which we prompted with a CLIP embedding of the object concept labels to further refine results. Images for which the segmentation algorithm failed (n=873) were excluded from this analysis. Next, we used object silhouettes identified through these segmentations as input to an image-computable model of object shape [9]. This model represents object shape with 22 dimensions, which have been demonstrated to be highly predictive of perceived shape similarity and which reflect latent components underlying more than 100 shape descriptors, such as fourier descriptors, major axis orientation, or shape skeleton [9]. If a given image contained multiple objects of the same type (e.g. 3 apples), we averaged by averaging over the values of all segmentations for this given image. From these results, we obtained an encoding model of object shape with 22 regressors. We then compared this shape model with our behavior-derived model in a variance partitioning analogous to our comparison with object category (see Methods *Variance partitioning of object category vs. dimension based models*). This allowed us to disentangle the amount of explained variance in neural responses that is uniquely attributable to object shape or the behaviorally-relevant dimensions, or that is shared by both.

Supplementary Methods 3: List of object categories and dimensions used in the variance partitioning

For the comparison of object categories and dimensions, we selected 50 superordinate categories and 30 dimensions.

The selected high-level categories included "animal", "bird", "body part", "breakfast food", "candy", "clothing", "clothing accessory", "condiment", "construction equipment", "container", "dessert", "drink", "electronic device", "farm animal", "food", "footwear", "fruit", "furniture", "game", "garden tool", "hardware", "headwear", "home appliance", "home decor", "insect",

"jewelry", "kitchen appliance", "kitchen tool", "lighting", "mammal", "medical equipment", "musical instrument", "office supply", "outerwear", "part of car", "plant", "protective clothing", "safety equipment", "school supply", "scientific equipment", "sea animal", "seafood", "sports equipment", "tool", "toy", "vegetable", "vehicle", "watercraft", "weapon" , and "women’s clothing".

The selected dimensions comprised "Metallic / artificial", "food-related", "animal-related", "textile", "plant-related", "house-related / furnishing-related", "valuable / precious", "transportation- / movement-related", "electronics / technology", "colorful / playful", "outdoors", "paper-related / flat", "hobby-related / game-related / playing-related", "tools-related / handheld / elongated", "fluid-related / drink-related", "water-related", "weapon-related / war-related / dangerous", "household-related", "feminine (stereotypical)", "body part-related", "music-related / hearing-related / hobby-related / loud", "construction-related / craftsmanship-related / housework-related", "spherical / voluminous", "flying-related / sky-related", "bug-related / non-mammalian / disgusting", "heat-related / fire-related / light-related", "foot-related / walking-related", "head-related", "medicine-related / health-related", and "sweet / dessert-related"

Supplementary Figures

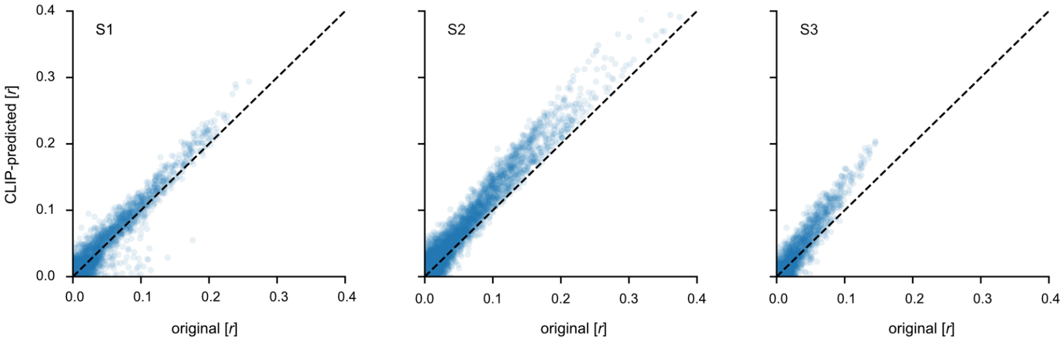

Supplementary Fig. 1. **Improvements in fMRI encoding model accuracy after image-wise prediction of object dimensions**. Scatter plots show the Pearson correlation between held out and predicted data (12-fold cross-validation) for our voxel-wise fMRI encoding model based on the 66 object dimensions underlying perceived similarity [5]. Each sample represents one voxel in a mask of visual cortex (V1-V3, FFA, OFA, pSTS, EBA, PPA, OPA, RSC). The x-axis denotes the prediction performance of the original object embedding based on 1,854 object concepts [5]. The y-axis denotes the prediction performance after these original object dimension weights have been predicted for each individual object image presented in fMRI (see Methods section on behavioral model). The dotted line shows equal performance in both cases.

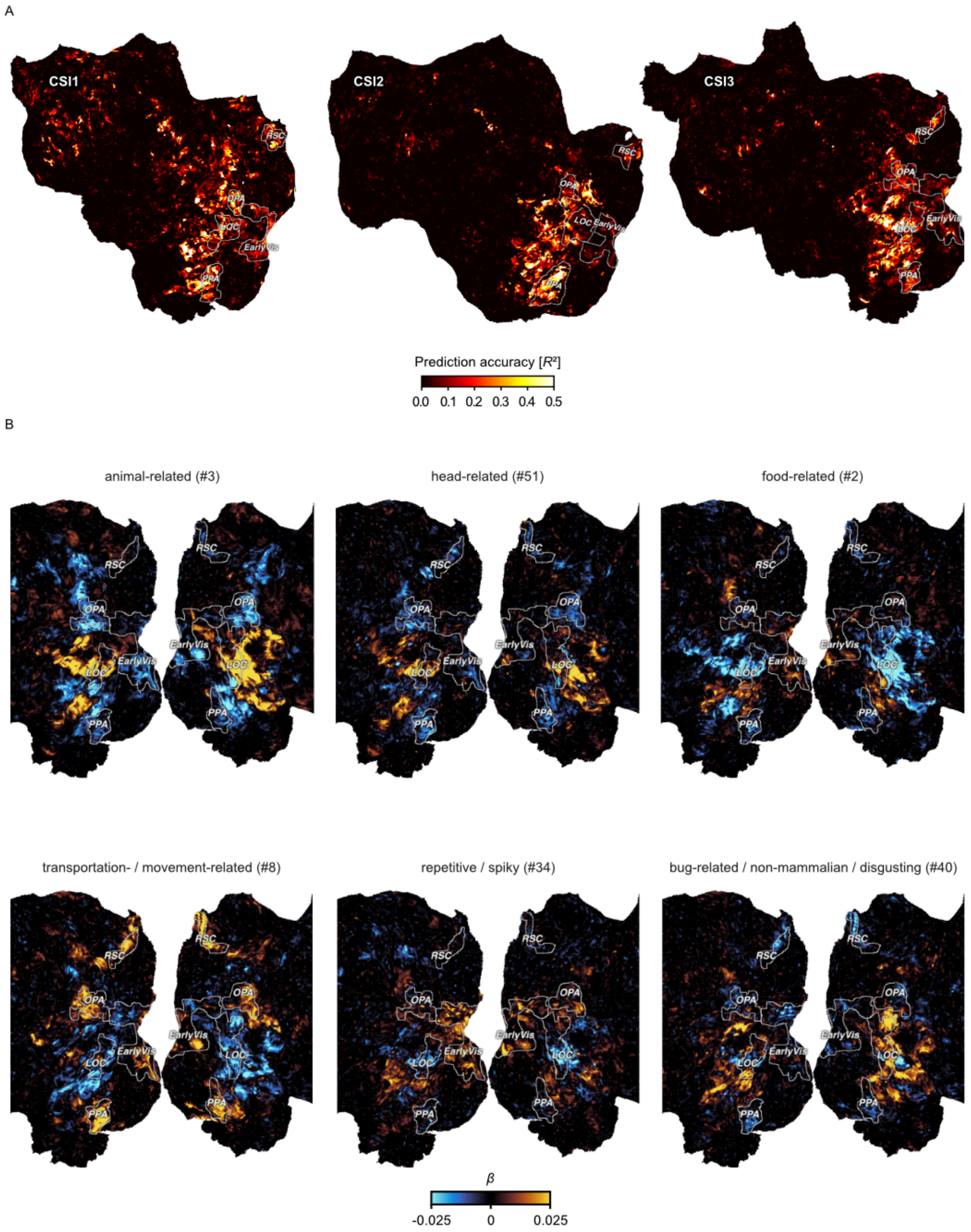

Supplementary Fig. 2. **Replication in the BOLD5000 dataset**. A. Prediction accuracy of an fMRI encoding model based on the predicted object embedding for BOLD5000 stimuli (noise-corrected R^2). Each column shows the flattened left cortical surface of one subject. The labels “CSI1”, “CSI2”, and “CSI3” correspond to the BOLD5000 subjects. Note that the ROI for early visual cortex is much smaller in BOLD5000 compared to the THINGS-fMRI dataset due to a smaller stimulus presentation (4.6 compared to 10 degree visual angle) and a different procedure for producing flat maps. B. Functional tuning maps for individual object dimensions in example subject CSI3.

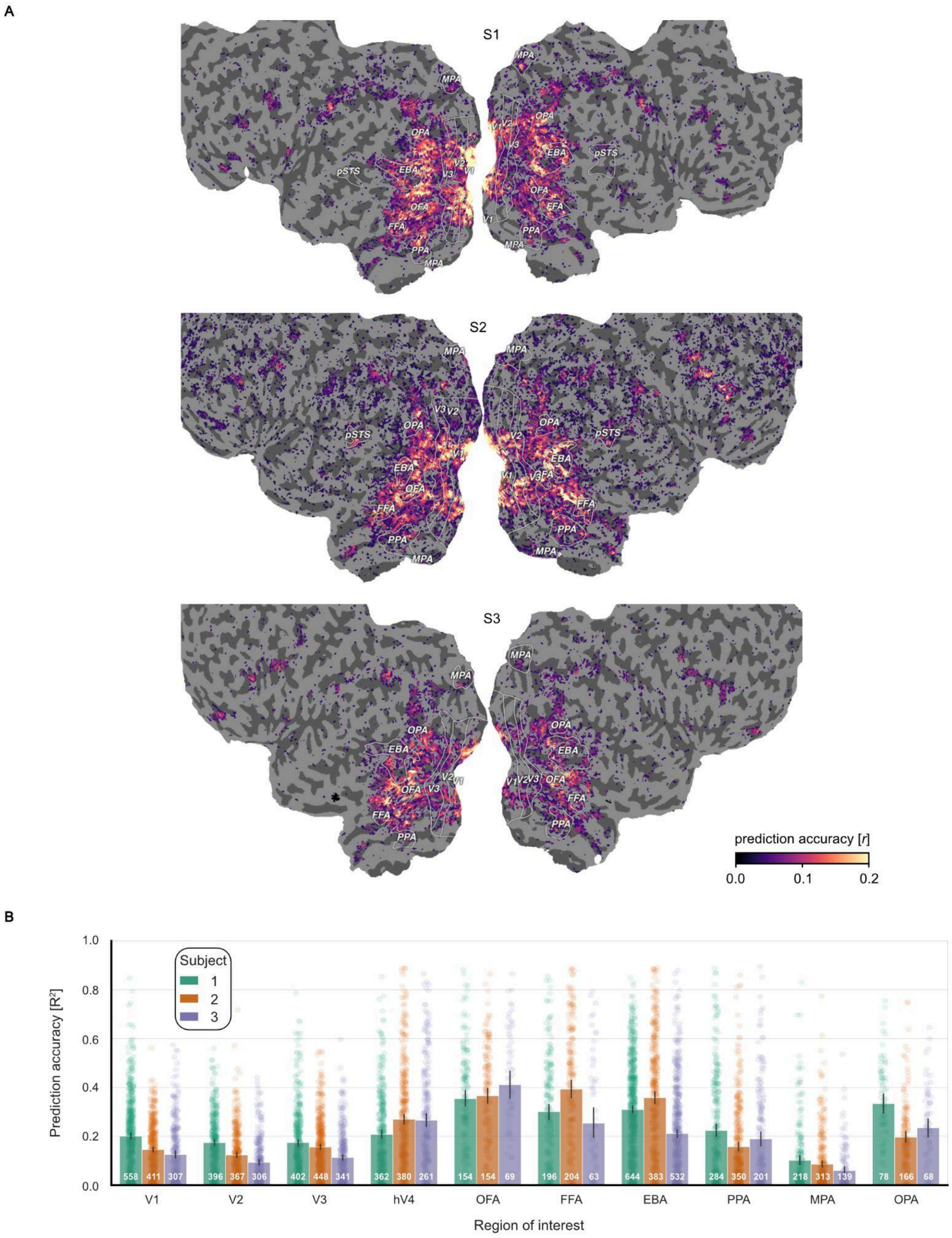

Supplementary Fig. 3. **fMRI encoding model prediction accuracy and average accuracy in different ROIs**. A. Prediction accuracy in statistically significant voxels (*p*<0.01, FDR-corrected, 12-fold cross-validation, 8,640 training and 820 test samples per fold, 10,000 random permutations per fold). Each row shows flattened cortical surfaces for each subject. Colors indicate Pearson correlation between predicted and held-out data in a between-session 12-fold cross-validation. B. Prediction accuracy in different regions of interest expressed as R^2. Regions of interest include retinotopic areas (V1, V2, V4, hV4) and category-selective clusters (OFA, FFA, EBA, PPA, MPA, OPA). Bars represent the mean value per ROI. Error bars indicate 95% confidence intervals of the mean. Each data point represents one voxel. White annotations indicate the number of voxels per ROI.

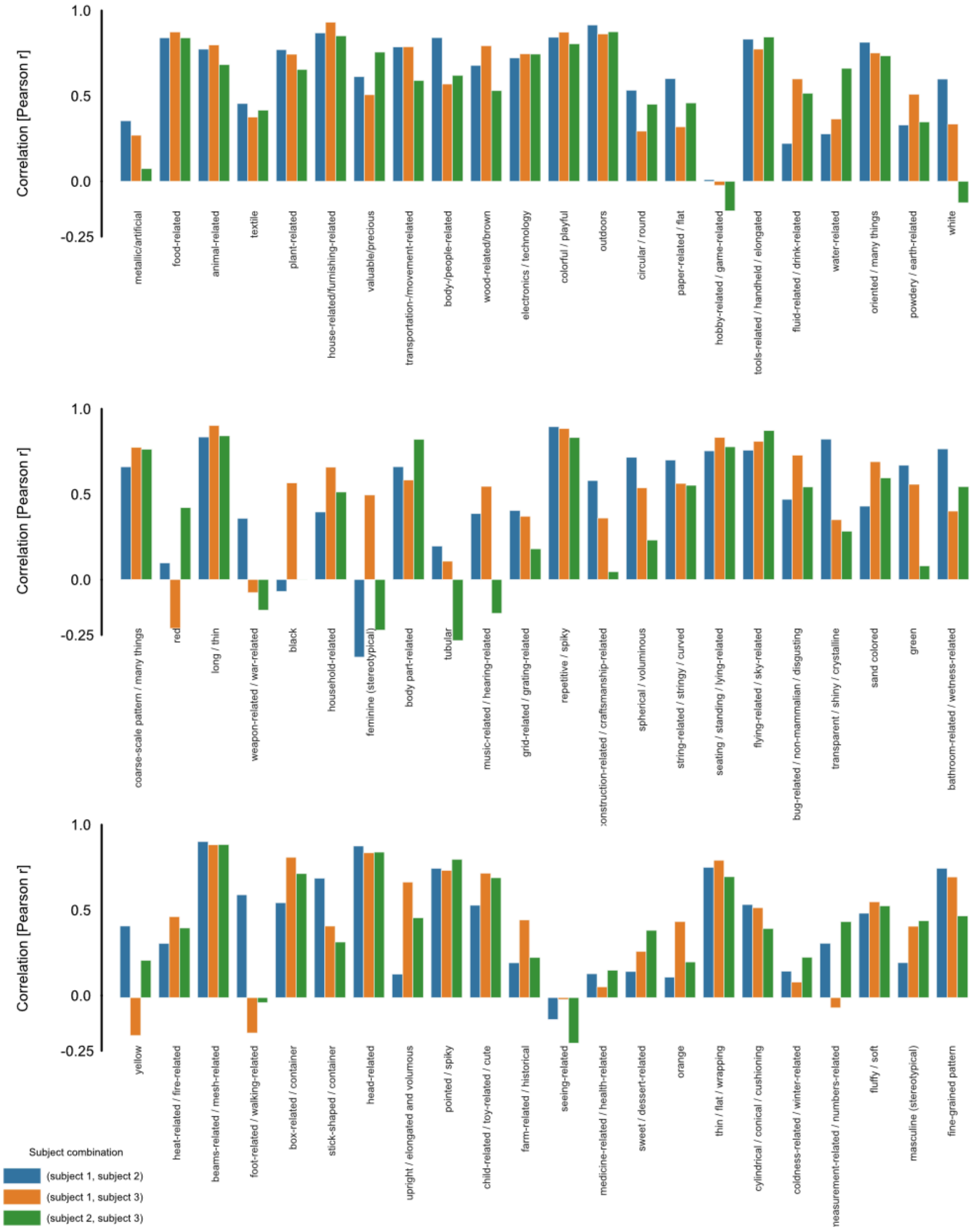

Supplementary Fig. 4. **Consistency of average ROI dimension tuning across subjects**. Bar heights show the correlation between two participants' dimension tuning patterns for a given dimension. Tuning patterns were obtained by averaging beta values from the encoding model in 16 ROIs. Bar color indicates the subject pair for which the correlation was computed.

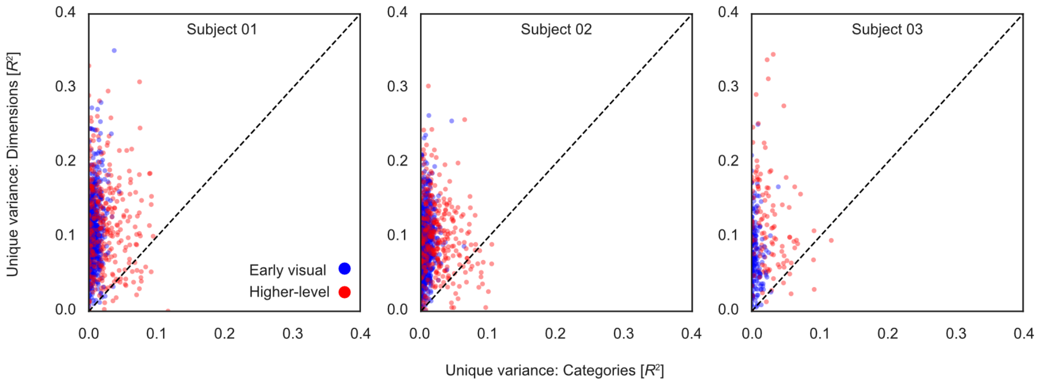

Supplementary Fig. 5. **Comparison of variance in neural responses uniquely explained by object category vs. dimensions**. Each sample represents one voxel. The x-axis indicates the amount of variance explained by an encoding model of object category, and the y-axis in turn by a model of behavior-derived dimensions. Voxels above the dashed identity line were better explained by the dimensions model. Color indicates whether voxels belong to early-visual (V1-V3) or higher-level (face-, body-, and scene-selective) regions of interest.

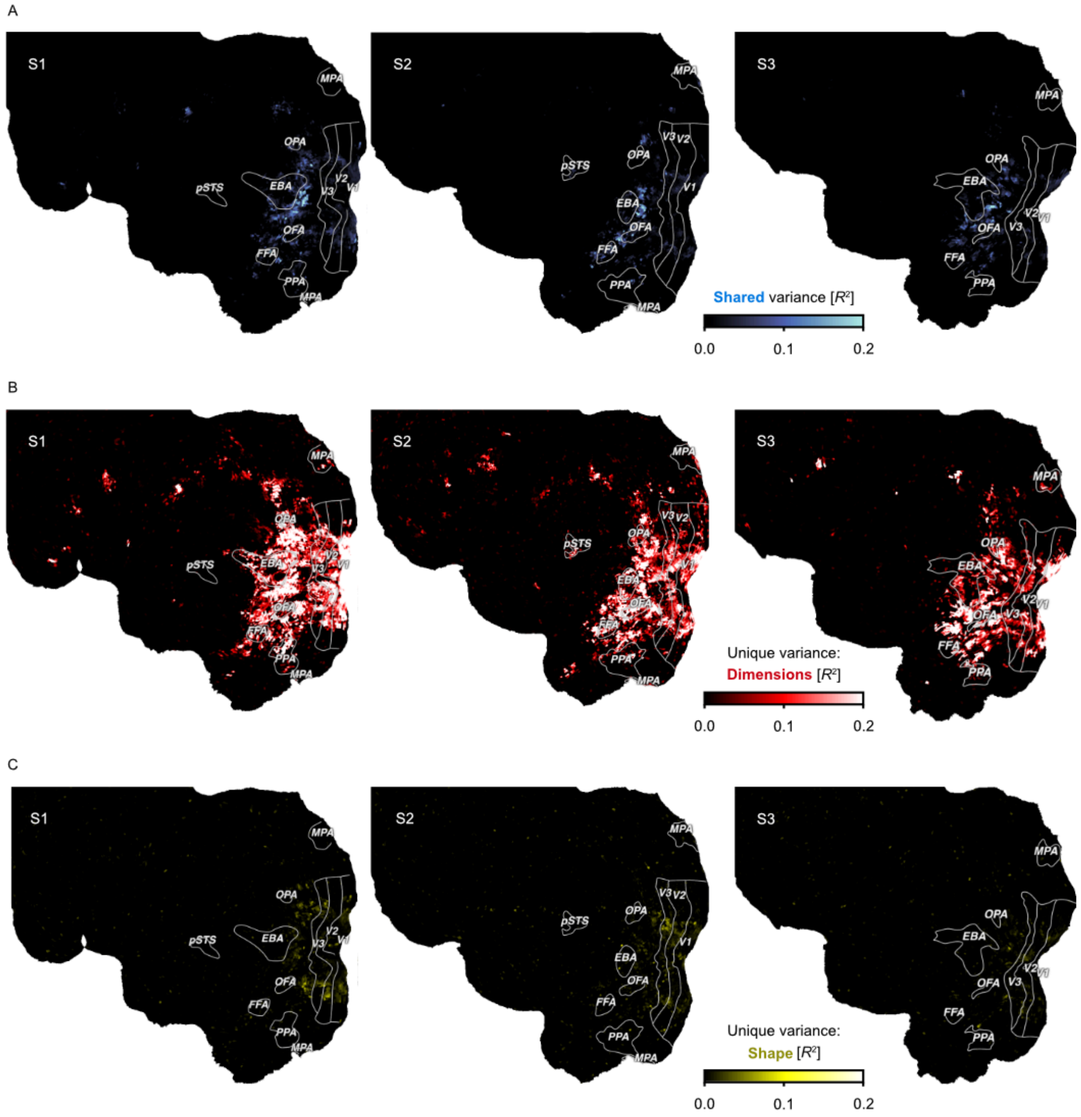

Supplementary Fig. 6. **Comparison of a model of object shape and the model of behavior-derived object dimensions.** Flat maps show the left hemisphere of each subject. Colors indicate the proportion of explained variance (noise ceiling corrected R^2) from variance partitioning. A. Shared variance explained by both models. B. Variance explained uniquely by the model of behavioral dimensions. C. Variance explained uniquely by a model of object shape.

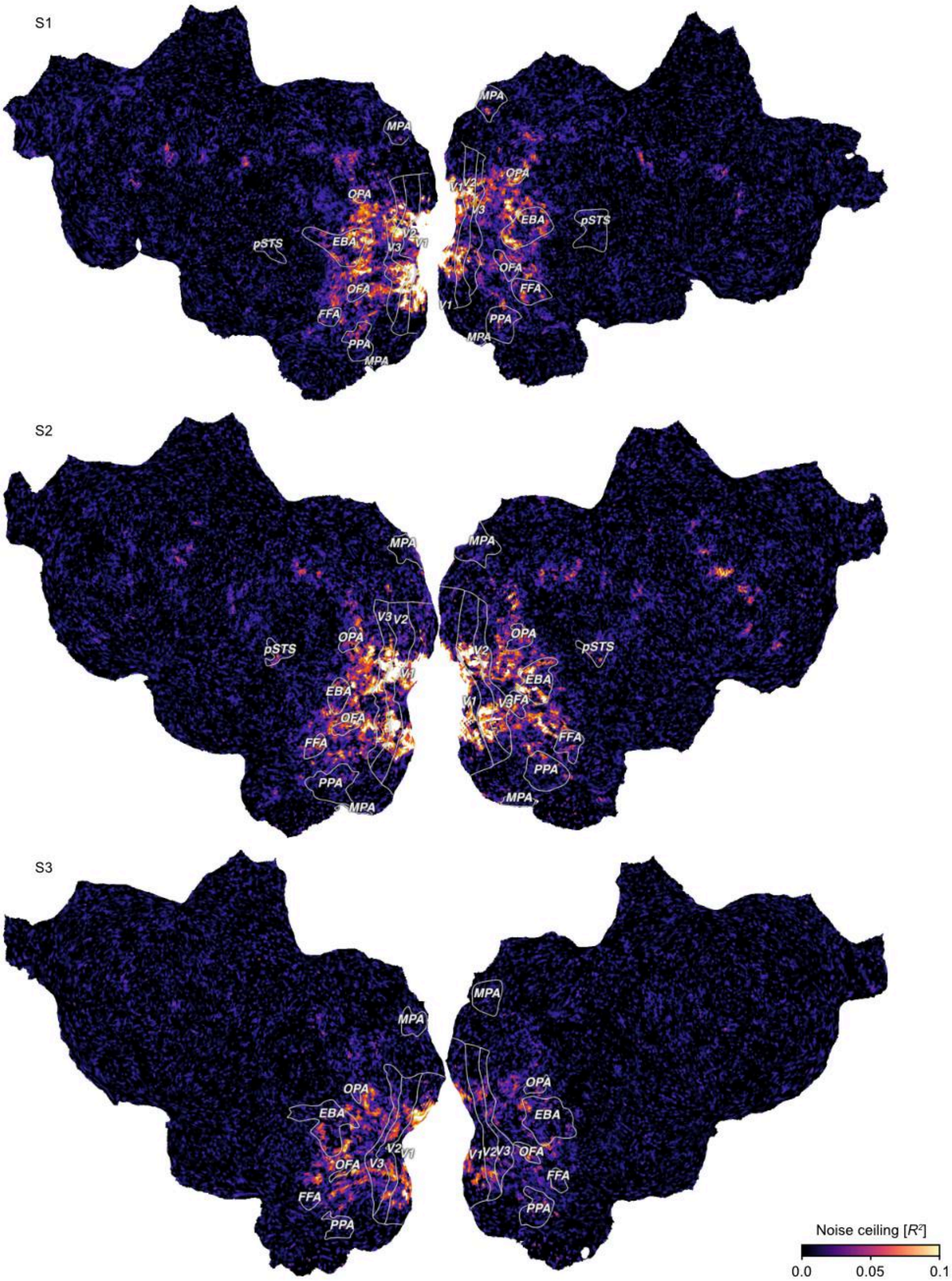

Supplementary Fig. 7. **Noise ceiling of single trial responses provided by the THINGS-fMRI dataset**. Colors indicate the noise ceiling expressed as the amount of explainable variance in trial-wise fMRI response estimates which was used to normalize the prediction performance of the encoding model.

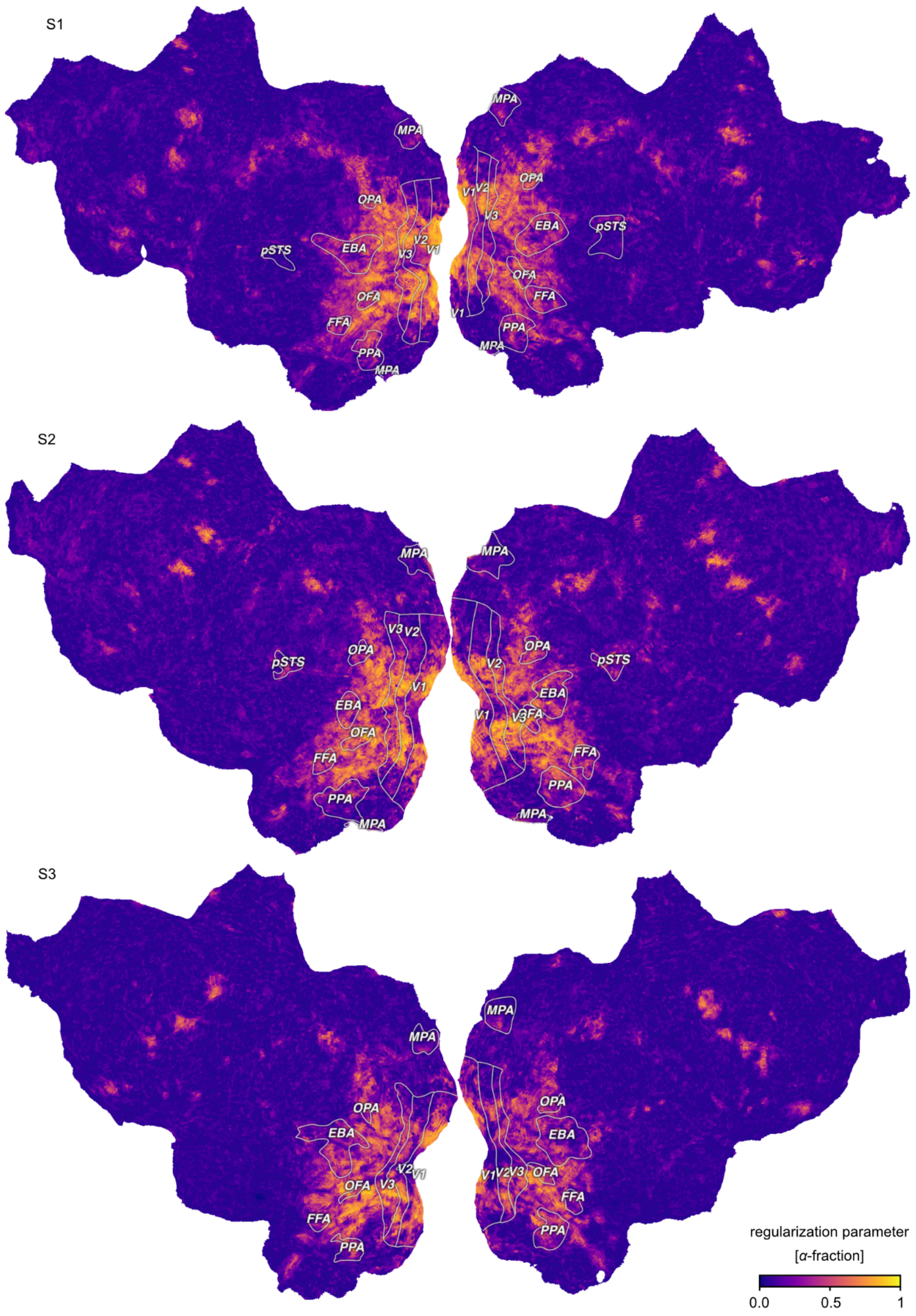

Supplementary Fig. 8. **Regularization parameter in the parametric modulation model**. Colors indicate voxel-wise α-fraction used to regularize the weights in the fractional ridge regression. Larger α-fraction reflect a smaller amount of regularization. An α-fraction of 1 is equivalent to the ordinary least squares solution. An α-fraction of 0 indicates maximum regularization, with all regression weights shrunk to 0.

References

1. Chang, N. *et al.* BOLD5000, a public fMRI dataset while viewing 5000 visual images. *Scientific Data* **6**, 49 (2019).
2. Deng, J. *et al.* ImageNet: A large-scale hierarchical image database. in *2009 IEEE Conference on Computer Vision and Pattern Recognition* 248–255 (IEEE, 2009).
3. Lin, T.-Y. *et al.* Microsoft COCO: Common Objects in Context. in *Computer Vision – ECCV 2014* 740–755 (Springer International Publishing, 2014).
4. Xiao, J., Hays, J., Ehinger, K. A., Oliva, A. & Torralba, A. SUN database: Large-scale scene recognition from abbey to zoo. in *2010 IEEE Computer Society Conference on Computer Vision and Pattern Recognition* 3485–3492 (IEEE, 2010).
5. Hebart, M. N. *et al.* THINGS-data, a multimodal collection of large-scale datasets for investigating object representations in human brain and behavior. *Elife* **12**, e82580 (2023).
6. Rokem, A. & Kay, K. Fractional ridge regression: a fast, interpretable reparameterization of ridge regression. *Gigascience* **9**, (2020).
7. Gao, J. S., Huth, A. G., Lescroart, M. D. & Gallant, J. L. Pycortex: an interactive surface visualizer for fMRI. *Front. Neuroinform.* **9**, 23 (2015).
8. Kirillov, A. *et al.* Segment Anything. *arXiv [cs.CV]* (2023).
9. Morgenstern, Y. *et al.* An image-computable model of human visual shape similarity. *PLoS Comput. Biol.* **17**, e1008981 (2021).
