# Supplementary material for: Distributed representations of behaviour-derived object dimensions in the human visual system
Source: Nat Hum Behav. 2024 Sep 9;8(11):2179–93. doi: 10.1038/s41562-024-01980-y (PMC11576512; doi:10.1038/s41562-024-01980-y)
Supplement: Supplementary file 2 — Reporting Summary [file 41562_2024_1980_MOESM2_ESM.pdf]

nature portfolio

Corresponding author(s): Oliver Contier

Last updated by author(s): Jun 27, 2024

Reporting Summary

Nature Portfolio wishes to improve the reproducibility of the work that we publish. This form provides structure for consistency and transparency in reporting. For further information on Nature Portfolio policies, see our Editorial Policies and the Editorial Policy Checklist.

Statistics

For all statistical analyses, confirm that the following items are present in the figure legend, table legend, main text, or Methods section.

n/a	Confirmed	
☐	☒	The exact sample size (*n*) for each experimental group/condition, given as a discrete number and unit of measurement
☐	☒	A statement on whether measurements were taken from distinct samples or whether the same sample was measured repeatedly
☐	☒	The statistical test(s) used AND whether they are one- or two-sided<br>*Only common tests should be described solely by name; describe more complex techniques in the Methods section.*
☒	☐	A description of all covariates tested
☒	☐	A description of any assumptions or corrections, such as tests of normality and adjustment for multiple comparisons
☐	☒	A full description of the statistical parameters including central tendency (e.g. means) or other basic estimates (e.g. regression coefficient) AND variation (e.g. standard deviation) or associated estimates of uncertainty (e.g. confidence intervals)
☐	☒	For null hypothesis testing, the test statistic (e.g. *F*, *t*, *r*) with confidence intervals, effect sizes, degrees of freedom and *P* value noted<br>*Give P values as exact values whenever suitable.*
☒	☐	For Bayesian analysis, information on the choice of priors and Markov chain Monte Carlo settings
☒	☐	For hierarchical and complex designs, identification of the appropriate level for tests and full reporting of outcomes
☐	☒	Estimates of effect sizes (e.g. Cohen's *d*, Pearson's *r*), indicating how they were calculated

Our web collection on statistics for biologists contains articles on many of the points above.

Software and code

Policy information about availability of computer code

Data collection	No additional data was collected for this manuscript. For a full description of the data acquisition including relevant computer software, see Hebart et al. 2023, https://doi.org/10.7554/eLife.82580
Data analysis	python (3.7.6) custom code with specified dependencies available at https://github.com/ViCCo-Group/dimension_encoding/

For manuscripts utilizing custom algorithms or software that are central to the research but not yet described in published literature, software must be made available to editors and reviewers. We strongly encourage code deposition in a community repository (e.g. GitHub). See the Nature Portfolio guidelines for submitting code & software for further information.

Data

Policy information about availability of data

All manuscripts must include a data availability statement. This statement should provide the following information, where applicable:

- Accession codes, unique identifiers, or web links for publicly available datasets
- A description of any restrictions on data availability
- For clinical datasets or third party data, please ensure that the statement adheres to our policy

The data supporting our analyses were obtained from the publicly available THINGS-fMRI dataset. The fMRI dataset is accessible on OpenNeuro (https://doi.org/10.18112/openneuro.ds004192.v1.0.5) and Figshare (https://doi.org/10.25452/figshare.plus.c.6161151). The object dimensions embedding underlying behavioral similarity judgements which was used to predict the fMRI responses is available at the Open Science Framework repository (https://osf.io/f5rn6/). The

higher-level object category labels which were used to construct a categorical model of object responses are part of the THINGSplus metadata and available at the Open Science Framework (https://osf.io/jum2f/). The BOLD 5000 data, including all images e.g. from the SUN database are openly available on figshare (https://doi.org/10.1184/R1/14456124).

Research involving human participants, their data, or biological material

Policy information about studies with human participants or human data. See also policy information about sex, gender (identity/presentation), and sexual orientation and race, ethnicity and racism.

Reporting on sex and gender	This study used already openly available data. No additional participants were recruited. More details can be found in the manuscript describing the data generation methods and consent information (https://elifesciences.org/articles/82580#s4).<br><br>2 of the 3 participants self-reported female gender. Neither sex nor gender was considered in study design. Neither sex- nor gender-related analyses were performed because the data, due to the small sample size, is unsuited for studying inter-individual effects. Participants had given consent for obtaining and sharing individual-level data.
Reporting on race, ethnicity, or other socially relevant groupings	No other socially relevant categorization variables were used in this manuscript.
Population characteristics	All participants were asked to report their age (Mean age at beginning of study: 25.33 years).
Recruitment	This study used already openly available data. No additional participants were recruited.
Ethics oversight	n/a

Note that full information on the approval of the study protocol must also be provided in the manuscript.

Field-specific reporting

Please select the one below that is the best fit for your research. If you are not sure, read the appropriate sections before making your selection.

☒ Life sciences ☐ Behavioural & social sciences ☐ Ecological, evolutionary & environmental sciences

For a reference copy of the document with all sections, see nature.com/documents/nr-reporting-summary-flat.pdf

Life sciences study design

All studies must disclose on these points even when the disclosure is negative.

Sample size	Analysis was performed on three subjects individually. The number of subjects in the open dataset we used is limited by the feasibility of data acquisition, which focused on densely sampled, large-scale recordings of neural responses for each individual subject instead of sampling a larger population.
Data exclusions	None of the THINGS-fMRI data had been excluded for this work. In the BOLD 5000 reanalysis, we excluded trials showing images from the SUN database because they did not contain objects.
Replication	We replicated our results in an independent dataset (BOLD5000), based on three different participants and different sets of stimuli (ImageNet and MS CoCo). All attempts at replication were successfull.
Randomization	Randomization did not apply to this work since we did not experimentally manipulate any variables. Instead, we reanalyzed already existing data.
Blinding	Blinding is not applicable to this work since we did not experimentally manipulate any variables.

Reporting for specific materials, systems and methods

We require information from authors about some types of materials, experimental systems and methods used in many studies. Here, indicate whether each material, system or method listed is relevant to your study. If you are not sure if a list item applies to your research, read the appropriate section before selecting a response.

Materials & experimental systems

n/a	Involved in the study
☒	☐ Antibodies
☒	☐ Eukaryotic cell lines
☒	☐ Palaeontology and archaeology
☒	☐ Animals and other organisms
☒	☐ Clinical data
☒	☐ Dual use research of concern
☒	☐ Plants

Methods

n/a	Involved in the study
☒	☐ ChIP-seq
☒	☐ Flow cytometry
☐	☒ MRI-based neuroimaging

Plants

Seed stocks

Report on the source of all seed stocks or other plant material used. If applicable, state the seed stock centre and catalogue number. If plant specimens were collected from the field, describe the collection location, date and sampling procedures.

Novel plant genotypes

Describe the methods by which all novel plant genotypes were produced. This includes those generated by transgenic approaches, gene editing, chemical/radiation-based mutagenesis and hybridization. For transgenic lines, describe the transformation method, the number of independent lines analyzed and the generation upon which experiments were performed. For gene-edited lines, describe the editor used, the endogenous sequence targeted for editing, the targeting guide RNA sequence (if applicable) and how the editor was applied.

Authentication

Describe any authentication procedures for each seed stock used or novel genotype generated. Describe any experiments used to assess the effect of a mutation and, where applicable, how potential secondary effects (e.g. second site T-DNA insertions, mosiacism, off-target gene editing) were examined.

Magnetic resonance imaging

Experimental design

Design type

Event-related task fMRI.

Design specifications

11,040 images (8,740 unique images, catch trials excluded, 500 ms presentation followed by 4 s of fixation). For details on the procedure of the fMRI and behavioral experiments, please consult the original publication of the dataset (https://elifesciences.org/articles/82580)

Behavioral performance measures

Participants responded to catch trials in order to stay engaged. Response accuracy was and catch trials were not analyzed.

Acquisition

Imaging type(s)

functional

Field strength

3

Sequence & imaging parameters

Gradient echo EPI, 2 mm isometric resolution, FOV = 192 mm × 192 mm, matrix size = 96 × 96; slice thickness: 2 mm, axial orientation, TR/TE/flip angle = 1.5s/33ms/75°

Area of acquisition

whole-brain

Diffusion MRI ☐ Used ☒ Not used

Preprocessing

Preprocessing software

The data used in this publication was already provided in preprocessed form. Additional smoothing (fwhm=4mm) was only performed for the sparseness analysis using the nilearn python library.

Normalization

Data were not normalized.

Normalization template

n/a

Noise and artifact removal

None

Volume censoring

None

Statistical modeling & inference

Model type and settings: Voxel-wise encoding model involving a cross-validated train-test procedure.

Effect(s) tested: Variance explained (r-squared) of the entire model.

Specify type of analysis: ☐ Whole brain ☐ ROI-based ☒ Both

Anatomical location(s): Object category-selective clusters were determined based on a standard functional localizer experiment. Similarly, retinotopic visual regions were determined based on a population receptive field experiment.

Statistic type for inference (See Eklund et al. 2016): voxel-wise

Correction: FDR

Models & analysis

n/a	Involved in the study
☒	☐ Functional and/or effective connectivity
☒	☐ Graph analysis
☐	☒ Multivariate modeling or predictive analysis

Multivariate modeling and predictive analysis: Independent variables: Object dimensions. Dependent variables: Voxel-wise responses to each object image. Average prediction performance was evaluated with a leave-one-session-out cross-validation and statistical significance was tested via permutation test (10,000 random permutations in each cross-validation fold, FDR $p<0.01$).
